# Persistence of antibodies in blood and body fluids in decaying fox carcasses, as exemplified by antibodies against *Microsporum canis*

**DOI:** 10.1186/1751-0147-48-10

**Published:** 2006-06-21

**Authors:** Morten Tryland, Kjell Handeland, Anna-Marie Bratberg, Inge-Tom Solbakk, Antti Oksanen

**Affiliations:** 1 The Norwegian School of Veterinary Science, Section of Arctic Veterinary Medicine, P.O. Box 6204, N-9292 Tromsø, Norway; 2 National Veterinary Institute, P.O. Box 8156 Dep., N-0033, Oslo, Norway; 3 National Veterinary and Food Research Institute EELA, Oulu Regional Unit, P.O.Box 517, FIN-90101 Oulu, Finland

## Abstract

To assist in evaluating serological test results from dead animals, 10 silver foxes (*Vulpes vulpes*) and 10 blue foxes (*Alopex lagopus*), 6 of each species previously vaccinated against and all challenged with *Microsporum canis*, were blood sampled and euthanased. Fox carcasses were stored at +10°C, and autopsy was performed on Days 0, 2, 4, 7, and 11 *post mortem *during which samples from blood and/or body fluid from the thoracic cavity were collected. Antibodies against *M. canis *were measured in an enzyme-linked immunosorbent assay (ELISA) as absorbance values (optical density; OD). To assess the degradation of antibodies, the ratio between *post mortem *and *ante mortem *absorbance was calculated. The mean absorbance from samples collected during autopsy was generally lower than from samples from live animals. In blood samples, this difference increased significantly with time (*P *= 0.04), while in body fluid samples the difference decreased (not significant; *P *= 0.18). We suggest that a positive serological result from testing blood or body fluid of a dead animal may be regarded as valuable, although specific prevalences obtained by screening populations based on this type of material may represent an under-estimation of the true antibody prevalence. Negative serological test results based on material from carcasses may be less conclusive, taken into account the general degradation processes in decaying carcasses, also involving immunoglobulin proteins.

## Background

Wild animal carcasses obtained for autopsy frequently are in an advanced stage of *post mortem *decomposition, which complicates morphological evaluation and impairs the possibility of making a proper diagnosis [1]. In addition to morphological examination, the wildlife pathologist may also utilise various tests, such as isolation of the infectious agent, detection of antigen in tissues by immunohistochemistry or polymerase chain reaction [2,3]. Also serological tests based on the demonstration of specific antibodies against various infective agents are performed on dead animals [4-6]. Thus, a systematic use of serological tests on autopsy material may give valuable information on the presence of specific infections in wildlife populations. The speed of *post mortem *decomposition of carcasses is affected by the ambient temperature, the higher the temperature, the faster the breakdown. The fate of immunoglobulins in decomposing carcasses is not well known, but obviously it will be subjected to decomposition as other proteins and organic matter. Systematic studies on the availability of blood and tissue fluids and their usability for serological testing during post mortem decomposition are missing. The present work was carried out to study the persistence of antibodies specific for the dermatophyte *Microsporum canis *in fox carcasses examined at different stages of *post mortem *decomposition.

At the end of a ringworm (*Microsporum canis*) vaccine-challenge trial in foxes [7], 10 apparently healthy farmed silver foxes (*Vulpes vulpes*) and 10 blue foxes (*Alopex lagopus*) were made available for this study. Six animals of each species had been vaccinated against *M. canis *(attenuated strains R 1/96 and R 2/96, National Veterinary Institute, Norway, strain collection, no adjuvance) at 4 and 6 weeks of age, and at the age of 11 weeks, all animals had been challenged by rubbing a suspension containing microconidia of a virulent strain of *M. canis *(strain R 14/96, National Veterinary Institute strain collection) topically on the back. Six weeks later, all foxes were anesthetised with xylazin (Rompun® Bayer AG, Leverkusen, Germany), blood sampled, and euthanased. Autopsy was performed using standard procedures. Four animals were autopsied 4 hours *post mortem *(Day 0), the remainder after storage for 2, 4, 7, 9, and 11 days (Table 1) at +10°C, which is about the mean summer temperature in the northern, sub-arctic part of Norway. At autopsy, the degree of decomposition was evaluated, and blood was collected after incising the base of the heart, hilus of the liver, and the femoral artery and vein. In addition, fluid from the thoracic cavity (body fluid) was collected when present. The blood and body fluid samples were centrifuged at 2800 g for 10 minutes, and the supernatant was collected and stored at -40°C until analysis.

**Table 1:** Measurements of antibodies against *Microsporum canis *in foxes. Mean and individual absorbance (optical density at 450 nm wavelength; OD) ratios at time of autopsy after different periods of storage at 10°C are presented.

Days *post mortem*	Animal id.	Species	Absorbance ratio	
			
			Blood *post mortem*/blood *ante mortem*	Body fluid *post mortem*/blood *ante mortem*
0	1	silver fox	0.75	n.a.
0	5	silver fox	0.79	0.18
0	16	blue fox	0.98	0.30
0	20	blue fox	0.88	n.a.
Mean ratio day 0:			**0.85**	**0.24**
2	2	silver fox	0.54	0.35
2	6	silver fox	0.76	0.08
2	11	blue fox	0.95	0.96
2	17	blue fox	1.19	n.a.
Mean ratio day 2:			**0.86**	**0.46**
4	7	silver fox	0.92	0.90
4	12	blue fox	0.38	0.39
Mean ratio day 4:			**0.65**	**0.64**
7	3	silver fox	n.a.	0.63
7	8	silver fox	n.a.	0.61
7	13	blue fox	0.74	0.70
7	18	blue fox	0.59	0.83
Mean ratio day 7:			**0.67**	**0.69**
9	9	silver fox	0.35	0.44
9	14	blue fox	0.34	0.58
Mean ratio day 9:			**0.35**	**0.51**
11	10	silver fox	n.a.	0.57
11	4	silver fox	n.a.	0.36
11	15	blue fox	n.a.	0.34
11	19	blue fox	n.a.	0.90
Mean ratio day 11:			-	**0.54**

n.a.= sample not available

**Figure 1:**
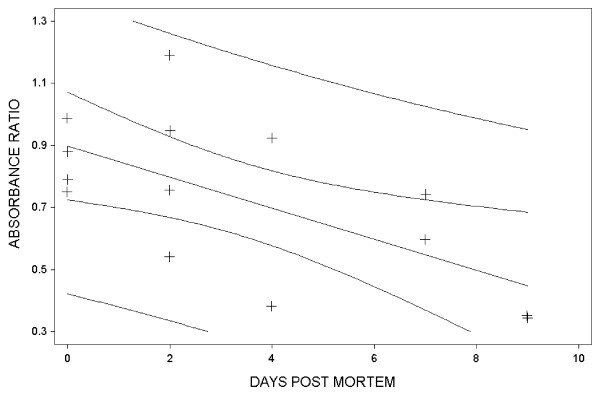
Linear regression plot for the relationship between the *post mortem *blood/*ante mortem *blood specific antibody ratio and time. Antibodies measured as absorbance (OD_450_). The lines surrounding the linear regression line indicate 95% confidence and prediction intervals.

**Figure 2:**
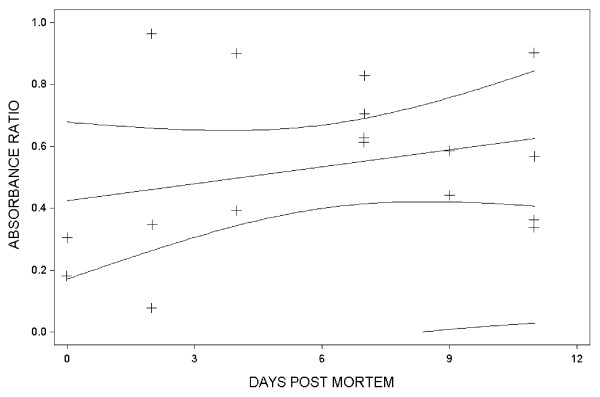
Linear regression plot for the relationship between the *post mortem *body fluid/*ante mortem *blood specific antibody ratio and time. Antibodies measured as absorbance (OD_450_). The lines surrounding the linear regression line indicate 95% confidence and prediction intervals.

Blood and body fluids were analysed by enzyme-linked immunosorbent assay (ELISA) used in the *M. canis *vaccination trial [7]. Immunoplates were coated with a soluble antigen (100 μl; 1 μg/ml) from microconidia of the *M. canis *strains used for vaccination. Serum samples were prediluted 1:4000 before tested. Rabbit-anti-silver fox IgG was used as secondary antibodies, whereas horseradish peroxidase-conjugated donkey anti-rabbit Ig was used as tertiary antibodies. Absorbance at 450 nm (OD_450_) was measured to express antibody concentration. To assess the *post mortem *development of absorbance from the day the foxes were killed to the day they were autopsied, the ratio between *post mortem *absorbance and *ante mortem *absorbance was calculated. The absorbance, with time, was used in a linear regression model (Statistix 7 for Windows software package; Analytical Software, Tallahassee, FL, USA). The absorbance ratios (*post mortem *blood/*ante mortem *blood, *post mortem *body fluid/*ante mortem *blood) of the samples from individual animals are given in Table 1. The *ante mortem *values were significantly higher in silver (mean 0.35, standard deviation (sd) 0.17) than in blue foxes (0.21, sd 0.06) (*P *= 0.02). The absorbance from samples collected during autopsy (both blood and body fluid) was generally lower than from samples from live animals. In blood samples, this difference increased significantly with time (*P *= 0.04), while in body fluid samples the difference decreased, but the decrease was not significant (*P *= 0.18). Plots for the regression models are shown in Figure 1 and 2.

Rigor mortis was present in all carcasses autopsied at day 0, and abundant amounts of fluid blood were available in the large vessels. Yellowish body fluid (< 1 ml) was obtained from the pericardial cavity of one animal. At day 2, rigor mortis had passed off. Considerable amount of fluid blood could be obtained from the large vessels, and a yellow to red body fluid (up to 1.5 ml) was available in the thoracic cavity of all carcasses. The carcasses examined at day 4 showed *post mortem *changes which included reduced intraocular tonus and greyish discoloured cornea, incipient yellow-green discoloration of the abdominal wall, and tissue imbibition. Moderate amounts of thick blood could be obtained from the large vessels, and yellow-red body fluid (up to 2 ml) was present in the thoracic cavity. One carcass also had a small amount of fluid in the pericardial cavity. In the carcasses examined at days 7 to 11, *post mortem *changes had progressed further. There was a grey-brown to green discoloration of the entire body wall. The lungs were brown-green discoloured, and the liver and kidneys had turned greyish and soft. At days 7–9 the carcasses had a nauseous odour, turning to decayed at day 11. Small amounts of thick blood were obtained from the large vessels of the carcasses at days 7–9. At day 11, only small amounts of semi-fluid blood were available. Several ml of red body fluid were present in the thoracic cavity at day 7. At days 9–11, the amount of fluid in the thoracic cavity had increased (up to 15 ml) and turned dark brown in colour.

We found generally higher absorbance values for silver foxes as compared to blue foxes. This may indicate a species dependent affinity, since rabbit-anti-silver fox IgG was used as secondary antibody in the ELISA. However, since we were interested in the difference in the absorbance from the day of euthanasia to the day of autopsy for each individual, the detected species difference in OD values should not represent a bias in this study. In the stored fox carcasses, fluid blood could be obtained from large vessels for about 1 week *post mortem*. The general and immediate decrease in absorbance, from *ante mortem *blood samples to *post mortem *blood samples on Day 0, obtained only 4 hours after death, was not expected, and we have no explanation for this. As could be expected due to post mortem breakdown of antibodies, the ratio between detectable antibodies in *post mortem *blood the following days and *ante mortem *blood was decreasing with time, indicating that the amount of antibodies against *M. canis *decreased in the blood of carcasses with the time of storage. Nine days *post mortem*, when some blood still was collectable, the mean absorbance had been substantially reduced by more than 50% in the current ELISA.

The amount of free body fluid in the thoracic cavity increased up to 11 days *post mortem *(the endpoint of the study). It was interesting and maybe unexpected to see that the ratio between detectable antibodies in post mortem body fluid and ante mortem blood increased with time, i.e. the amount of anti-*M. canis *antibodies in body fluids increased slightly with the time of storage of the foxes. This may indicate that the body fluid compartment became more concentrated over time in spite of increasing in volume, possibly due to a general dehydration of the carcasses. By practical means, it seems that when the availability of liquid blood decreases, sampling and testing body fluids may be a reasonable alternative. It is also possible that other types of tissue fluids would be as well or better suited for demonstrating antibodies than blood or the free body fluid used in the present trial [8]. As an example, muscle fluid has been shown to be suitable in serology studies on *Trichinella *sp. in swine [9].

In conclusion, we suggest that a positive serological result from blood or body fluid of a dead animal may be regarded as valuable, indicating exposure to specific infectious agents even many days post mortem. A negative test result, however, may be less conclusive. Hence, specific seroprevalences obtained from a screening of a population based on blood or body fluids from carcasses may represent an under-estimation of the true prevalence in the population. Body fluid from the thoracic cavity seems to constitute an alternative testing material when blood is no longer suited or available in the carcass.
